# Case Report: Afatinib Treatment in a Patient With NSCLC Harboring a Rare *EGFR* Exon 20 Mutation

**DOI:** 10.3389/fonc.2020.593852

**Published:** 2021-01-26

**Authors:** Sabine Zöchbauer-Müller, Bettina Kaserer, Helmut Prosch, Agnieszka Cseh, Flavio Solca, Markus Johann Bauer, Leonhard Müllauer

**Affiliations:** ^1^ Clinical Division of Oncology, Department of Medicine I, Medical University of Vienna, Vienna, Austria; ^2^ Comprehensive Cancer Center, Vienna, Austria; ^3^ Institute of Pathology, Medical University of Vienna, Vienna, Austria; ^4^ Department of Biomedical Imaging and Image-Guided Therapy, Medical University of Vienna, Vienna, Austria; ^5^ Boehringer Ingelheim International GmbH, Ingelheim, Germany; ^6^ Boehringer Ingelheim RCV GmbH & Co. KG, Vienna, Austria

**Keywords:** afatinib, *EGFR* mutation, exon 20 insertion, H773dup, long-term response, NSCLC, uncommon mutation

## Abstract

Unlike most other primary epidermal growth factor receptor (*EGFR*) mutations in non-small cell lung cancer (NSCLC), exon 20 insertions, comprising approximately 4% to 10% of all *EGFR* mutations, are generally considered to be resistant to EGFR tyrosine kinase inhibitors (TKIs). However, *EGFR* exon 20 insertions are structurally and pharmacologically heterogeneous, with variability in their position and size having implications for response to different EGFR TKIs. The second-generation ErbB family blocker, afatinib, is approved for the first-line treatment of *EGFR* mutation-positive NSCLC and has been shown to have a broad inhibitory profile against common and uncommon *EGFR* mutations. Here, we describe a patient with bilateral multifocal lung adenocarcinoma harboring a very rare *EGFR* exon 20 insertion (c.2317_2319dup3; p.H773dup), who has been receiving treatment with afatinib for 4.5 years. To our knowledge, this is the first report describing long-term benefit for a patient treated with afatinib with this rare exon 20 insertion. We are aware of two further cases with this rare *EGFR* mutation. One patient, also reported here, has early-stage lung adenocarcinoma and has not yet received systemic therapy for NSCLC. The other patient received afatinib in the context of a global compassionate use program and had progressive disease. Our findings may be of clinical relevance for patients carrying tumors with this rare mutation as epidemiological evidence suggests that p.H773dup may function as a driver mutation in NSCLC. Together with previous preclinical and clinical evidence for the activity of afatinib against certain *EGFR* exon 20 insertions, these findings warrant further investigation.

## Introduction

In non-small cell lung cancer (NSCLC), activating mutations in the epidermal growth factor receptor (*EGFR*) gene are reported in approximately 10% to 15% of Caucasian and 50% of Asian patients (1). *EGFR* mutation-positive tumors tend to be dependent on EGFR signaling for their growth and survival and, consequently, several EGFR-targeted therapeutics have been developed and approved. Two types of *EGFR* mutation, exon 19 deletions (Del19) and the exon 21 substitution L858R, represent approximately 45% and 40% of all *EGFR* mutations, respectively (2). Del19 and L858R are therefore classed as common *EGFR* mutations and are the best characterized in terms of their association with response to EGFR TKIs (3). For these mutations, drugs approved by the United States (US) Food & Drug Administration (FDA) for *EGFR* mutation-positive NSCLC comprise three generations of EGFR tyrosine kinase inhibitors (TKIs) — the first-generation reversible EGFR TKIs, erlotinib and gefitinib, the second-generation irreversible ErbB family blockers, afatinib and dacomitinib, and the third-generation irreversible, EGFR wild-type sparing TKI, osimertinib (4).

Many of the less common mutations are also sensitive to EGFR inhibitors. Afatinib, in particular, shows a broad preclinical activity across uncommon *EGFR* mutations (5), and has demonstrated clinical efficacy against uncommon mutations such as G719X, S768I, and L861Q (in exons 18, 20, and 21, respectively) (6). Based on these findings, the US indication for afatinib was extended to include S768I, L861Q and G719X mutations (7), while all activating *EGFR* mutations were already included in the labels in Europe (8).

Treatment options for the most prevalent uncommon mutations, i.e. *EGFR* exon 20 insertions (~4–10% of all *EGFR* mutations), are not clear and represent an area of unmet need. A recent Phase 2 study, ZENITH20, assessed poziotinib, a covalent inhibitor of EGFR and human epidermal growth factor receptor 2 (HER2), in pretreated NSCLC patients with exon 20 insertions. Although the study did not meet its primary endpoint, there was some evidence of clinical activity, with an objective response rate (ORR) of 15%, disease control rate of 69% and median progression-free survival (PFS) of 4.2 months. Treatment-naïve cohorts using alternative dosing regimens to improve tolerability are ongoing (9). Other investigational agents, including the EGFR/HER2 inhibitor, TAK-788 (10) and the bispecific EGFR/cMET antibody, JNJ-372 (11), are in clinical development. While, in principle, most *EGFR* exon 20 insertions could be considered as oncogenic driver mutations, since they promote interleukin 3- and EGF-independent growth of Ba/F3 cells (5), structural and pharmacological differences between specific mutations mean that their sensitivity to targeted treatment differs depending on the inhibitor used and the mutational context. Some exon 20 insertions exist as compound mutations, which could also contribute to EGFR-TKI resistance (5). With the exception of A763_Y764insFQEA (12), most *EGFR* exon 20 insertion mutations are resistant to first-generation EGFR TKIs, while the efficacy of second-/third-generation TKIs against these mutations is less clear (13). Indeed, a post-hoc analysis of the LUX-Lung 2, 3, and 6 trials suggested limited activity of afatinib treatment in patients with *EGFR* exon 20 insertions (6). In 23 such patients the ORR was 8.7% and median PFS was 2.7 months (6). In contrast, data from the afatinib uncommon *EGFR* mutations database indicate that afatinib has modest but apparent clinical activity. The response rate in 70 TKI-naïve patients with exon 20 insertions was 24% and median duration of response was 11.9 months (14). In addition to the mutations known to be responsive to EGFR TKIs, a number of rare exon 20 insertions showed sensitivity to afatinib including A767delinsASVD (13) and A767_S768insSVA (15).

**Figure 1:**
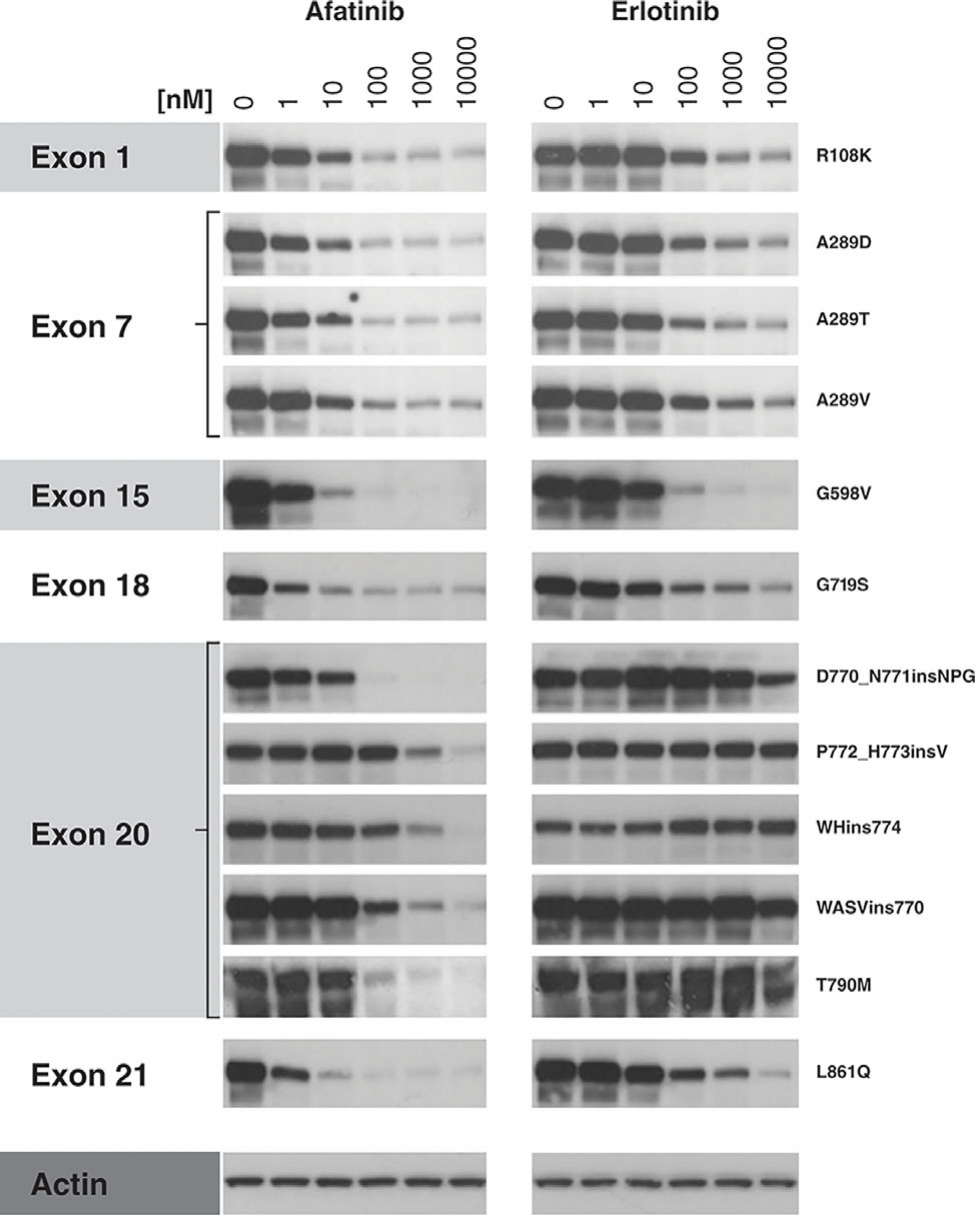
Inhibition of *EGFR* mutant protein autophosphorylation by afatinib and erlotinib in cellular assays. NIH-3T3 cells (ATCC; #CRL-1658) were cultured in supplemented Dulbecco’s Modified Eagle Medium and cultivated at 37°C/5% CO_2_ in a humidified atmosphere to maintain <80% confluence. Cells were then transfected with one of 12 *EGFR* mutant plasmid constructs, using 4 µg of DNA for EGFR variants G598V, D770_N771insNPG, P772_H773insV, WHins774, and T790M, and 2.5 µg of constructs R108K, A289D, A289T, A289V, G719S, WASVins770, and L861Q, diluted in 250 µl serum-free culture medium. A diluted Lipofectamine-DNA mix was then added drop-wise to the cells. 48 h post-transfection, cells were treated for 2 h with afatinib or erlotinib (1–10,000 nM) or were left untreated. At 2 h post-treatment, protein lysates were prepared using lysis buffer and the effect of TKI treatment on EGFR tyrosine-1068 phosphorylation was analyzed by Western blot (primary antibody: phospho-specific anti-EGFR [Y1068] antibody; Abcam, #ab40815, 1:1,000 dilution; secondary antibody: goat anti-rabbit; Dako, #P0448, 1:1,000 dilution). Actin was used as a loading control. *EGFR*, epidermal growth factor receptor.

Previously published preclinical evidence shows that afatinib displays inhibitory activity against some *EGFR* exon 20 insertion mutations (5, 16–18). Using ectopically expressed *EGFR*-mutants in NIH-3T3 cell lines, we found that, consistent with previous findings, afatinib was at least 100-fold more potent against G719S (Exon 18) and L861Q (Exon 21) mutations (**Figure 1**) compared to erlotinib. For most exon 20 mutations, EGFR phosphorylation was inhibited by afatinib at concentrations exceeding the clinically relevant C_max_ of 100 nM (except D770_N771insNPG), while erlotinib was ineffective in reducing constitutive phosphorylation in the exon 20 mutations tested at concentrations up to 10000 nM (**Figure 1**). The observed biomarker modulation data is in-line with previously reported proliferation data in the BA/F3 system (IC_50_ of afatinib for Y764_V765insHH, A767_V769dupASV, and D770_N771insNPG were 134, 158, and 43 nM, respectively) (10, 16). Taken together, these data show differential sensitivity of EGFR exon 20 mutations to different EGFR-TKIs in preclinical and clinical contexts. Careful evaluation of the EGFR mutational context, including potency and therapeutic window, will be essential to select appropriate treatments for patients harboring tumors with EGFR exon 20 mutations.

Here, we report on a case of a patient with a very rare *EGFR* exon 20 insertion (c.2317_2319dup3; p.H773dup) who has been receiving treatment with afatinib for 4.5 years. In addition, we describe a patient in the early stages of lung cancer treatment, who underwent surgery at the same institution and had an identical p.H773dup mutation.

## Case Report 1

**Figure 2:**
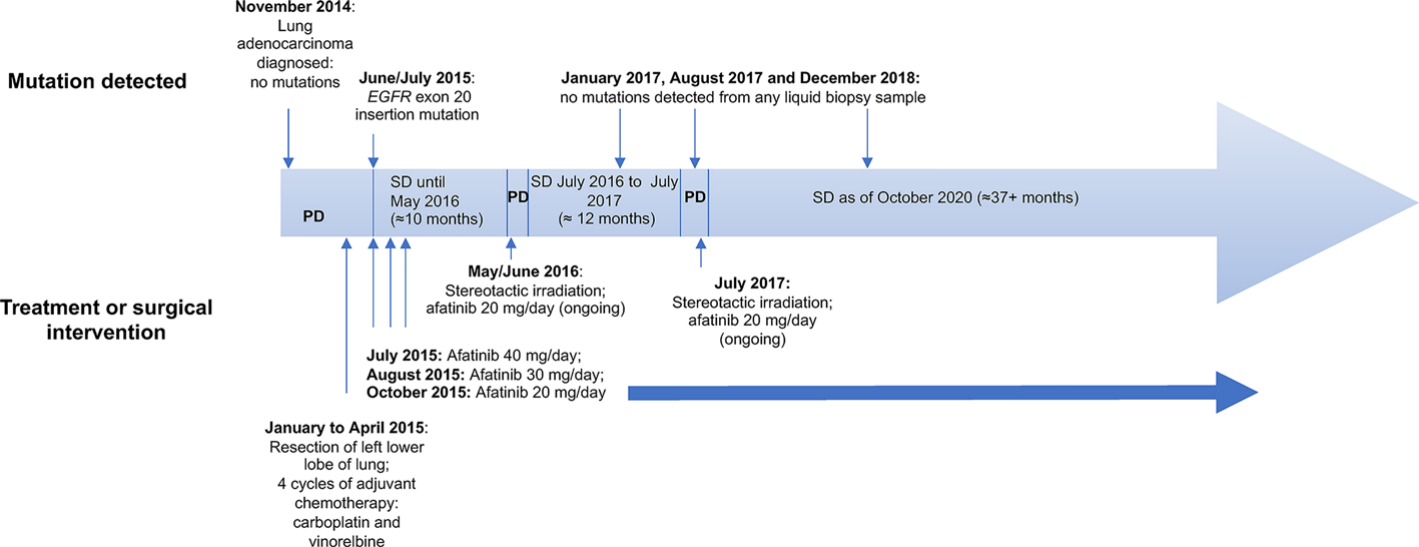
Patient 1 case history and time line of key events. PD, progressive disease; SD, stable disease.

Patient 1 was a 62-year-old, female, ex-smoker (20 pack-years) with no major comorbidities. She was incidentally diagnosed in November 2014 with a 6.4 cm mass in the left upper lobe and multiple ground glass nodules in both lungs, the largest of which was located in the right middle lobe, with a maximum diameter of 1.8 cm. Histopathology following resection of the left upper lobe confirmed lung adenocarcinoma (Grade 2; pT2b pN1 [2/17 lymph nodes positive]; V0 R0), with partially papillary and partially tubular morphology. Molecular pathology based on Sanger sequencing of *EGFR* exons 18 to 21 indicated no *EGFR* mutation, and the tumor was *ALK* fluorescence *in situ* hybridization negative and ROS1 immunohistochemistry-negative (**Figure 2**).

**Figure 3:**
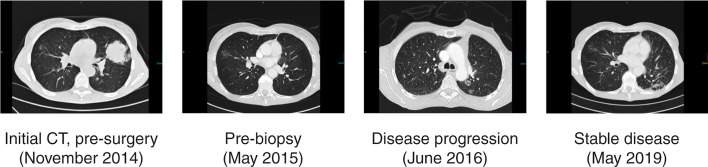
Patient 1 clinical course, including treatment history and CT scans. CT, computed tomography.

The patient received 4 cycles of adjuvant chemotherapy with carboplatin and vinorelbine from January 2015 until April 2015, but a computed tomography (CT) scan in May 2015 showed disease progression. The scan revealed several pulmonary focal ground-glass opacities (GGOs) in both lungs, some of which had increased in size compared with preoperative CT findings (**Figure 3**). Biopsy of a GGO lesion of the right upper lobe in June 2015 indicated Grade 2 adenocarcinoma of the lung that was partially tubular and partially lepidic.

**Figure 4:**
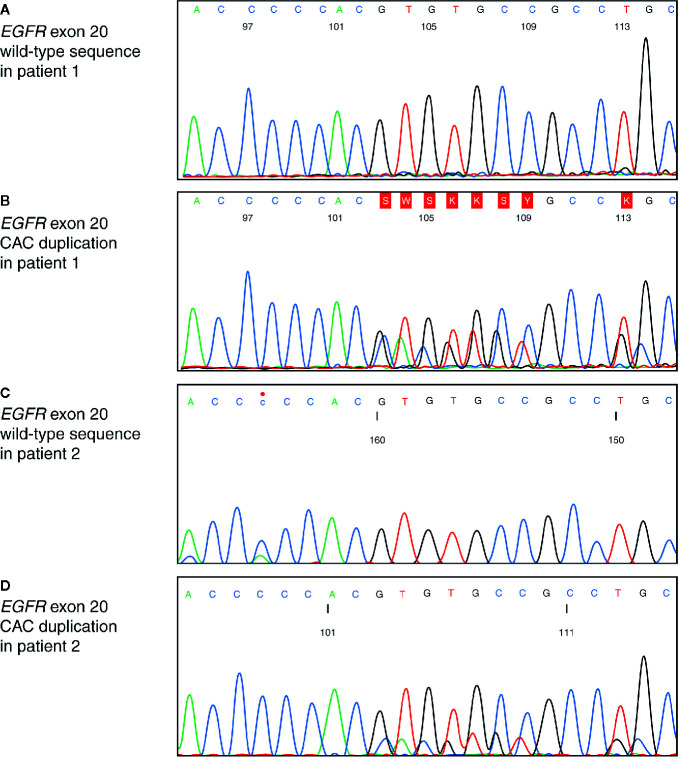
Insertion site of *EGFR* exon 20 insertion mutation. Sequencing electropherogram of *EGFR* exon 20 showing: **(A)** wild-type sequence of the adenocarcinoma from patient 1 resected in 2014; **(B)** duplication of a CAC base triplet in the adenocarcinoma from patient 1 biopsied in 2015. *EGFR*, epidermal growth factor receptor; CAC, cytosine adenine cytosine; **(C)** wild-type sequence of the adenocarcinoma from patient 2 resected in 2019; **(D)** duplication of a CAC base triplet in the adenocarcinoma from patient 2 biopsied in 2019.

Further Sanger sequencing detected an *EGFR* exon 20 insertion mutation (NM_005228.3 [EGFR]: codon 2317_2319dupCAC; p.H773dup; **Figures 4A, B** **)**. From July 2015, the patient received afatinib 40 mg/day, which was reduced to 30 mg/day in August 2015 and further reduced to 20 mg/day in October 2015 due to diarrhea. The patient achieved stable disease until May 2016, followed by progression, which was treated with stereotactic irradiation to two lesions (40.5 Grays in 3 fractions to each lesion) in the left lower lobe and one lesion in the right upper lobe. She remained on treatment with afatinib (20 mg/day) and had stable disease again from July 2016 until July 2017 (**Figure 3**), when progression ensued and she received stereotactic irradiation to one lesion in the left lower lobe.

Two liquid biopsies were performed, in January and August 2017. No *EGFR* or any other mutation was detected in either sample, using next-generation sequencing (NGS) with a colon/lung 22-gene (including *EGFR*) panel from Thermo Fisher Scientific. The sample from January was additionally analyzed with digital PCR for Del19 and T790M mutations, with none detected. In December 2018, a third liquid biopsy was performed, this time employing an NGS liquid biopsy lung circulating free DNA (11-gene) panel from Thermo Fisher Scientific. Again, no mutations were detected.

Ileocecal resection was performed in April 2018 because of ischemic necrosis of the cecum, and no carcinoma infiltration was detected in the resection specimen. Because of slightly enlarged retrocaval lymph nodes in a CT scan from December 2018, lymph node and lung biopsies were performed in January 2019. Endobronchial ultrasound-guided biopsy of the mediastinal lymph nodes revealed no carcinoma infiltration, and bronchoscopy with biopsy lower right lobe (B10) identified no carcinoma in the lung parenchyma. However, *Escherichia coli* and *Klebsiella pneumonia* were detected in the bronchoalveolar lavage, and the patient received antibiotic treatment with ciprofloxacin. A CT scan in February 2019 showed a decrease in size of the previously enlarged lymph nodes. Treatment with afatinib is ongoing as of October 2020, and the patient continues to have stable disease (**Figure 3**), with an Eastern Cooperative Oncology Group performance status of 0.

## Case Report 2

Patient 2 was referred to our clinic for surgery in September 2019. The patient had previously undergone resection of breast carcinoma, and received radiotherapy and hormone ablation therapy. A lung tumor was detected with position emission tomography-CT scan, and resection of the left lower lobe with lymphadenectomy was performed in September 2019, due to suspicion of lung metastasis of the breast carcinoma. Histology revealed invasive adenocarcinoma, and NGS with the Oncomine Focus Assay (Thermo Fisher Scientific) identified an *EGFR* exon 20 insertion (NM_005228.3 [EGFR]: codon 2317_2319dupCAC; p.H773dup; **Figures 4C, D**) that was identical to the mutation identified in Patient 1.

After tumor resection, the patient was re-transferred to their previous hospital.

## Discussion and Concluding Remarks

Exon 20 insertion mutations are the third most common type of *EGFR* mutation, after Del19 and L858R (3). Similar to Del19 and L858R, *EGFR* exon 20 insertions can result in sustained EGFR signaling and function as oncogenic drivers. However, despite their importance as potentially targetable mutations, the clinicopathologic characteristics and molecular spectrum of exon 20-mutant tumors have not been explored in most patient populations, and the biology underlying the heterogeneous responses of different genomic variants to targeted therapies is not well understood (3).

The first case presented herein describes a patient with NSCLC harboring an *EGFR* exon 20 insertion mutation who achieved durable stable disease with afatinib and remains on treatment after 4.5 years. To the best of our knowledge, this is the first report describing long-term benefit for a patient treated with afatinib with this rare exon 20 insertion. We are aware of two further cases with this same, rare *EGFR* mutation. One patient, also reported here, has early-stage lung adenocarcinoma and has not yet received systemic therapy for NSCLC. The other patient received afatinib in the context of a global compassionate use program and had progressive disease (19, 20). It is not uncommon for patients to have concurrent mutations alongside exon 20 insertions (21); therefore, we speculate that the difference in afatinib response could be owing to the presence of an additional mutation. For example, TP53 mutations are one of the most common concurrent mutations alongside exon 20 insertion (21) and is possibly associated with a lower likelihood of response to EGFR TKIs (22). Overall, these findings are particularly interesting and suggest that afatinib may provide a new therapeutic option for the particular type of mutation discussed here.

The exon 20 H773dup insertion, annotated as H773_V774insH, has been reported previously, although such reports have not provided evidence for a potential driver role for this particular mutation (3, 23). To determine its prevalence in current databases, we searched for the occurrence of H773dup within the American Association for Cancer Research (AACR) Genomics Evidence Neoplasia Information Exchange (GENIE) database (The AACR Project GENIE Consortium, release 5.0), which comprises almost 60,000 samples across 81 major cancer types (24). We found 15 cases of H773dup in NSCLC, glioma, and endometrial cancer, showing prevalences of 0.12%, 0.06%, and 0.12%, respectively (**Table 1A** **)**. In line with Qin et al. (21), we also find co-mutations in cancer-related genes like TP53 (4/15), PIK3CA (1/15), or PTEN (1/15) which might contribute to different responses upon treatment with TKIs. As a second source, we queried FoundationCore (version MI20190726), a proprietary database provided by Foundation Medicine. FoundationCore contains almost 300,000 clinical specimens and represents, to our knowledge, the biggest available database of its kind, allowing us to exhaustively describe the H773dup mutation landscape. In line with the GENIE results, we found H773dup mutations in NSCLC (adenocarcinoma and squamous cell carcinoma) and glioma (glioblastoma and anaplastic astrocytoma) at a prevalence of 0.1%, as well as in other (niche) indications. **Table 1B** lists all instances of H773dup, highlighting its widespread occurrence. In addition to mutation information, FoundationCore provides copy-number information for all samples. Interestingly, 33% and 43% of lung adenocarcinomas of glioblastomas, respectively, show co-occurring *EGFR* amplifications, which are indicative of H773dup having a functional role. This corroborates an earlier claim that *EGFR* exon 20 insertions may function as driver mutations that are potentially susceptible to effective targeted therapy, based on exon 20 insertions being enriched in never-smokers and Asian patients (23).

**Table 1:** Occurrence of H773dup mutations in the AACR Project GENIE and FoundationCore databases.

A) AACR Project GENIE				
Cancer Type	Total number of samples	Number of H773dup mutations	Prevalence (%)	95% CI
NSCLC	9090	11	0.121	0.060–0.216
Glioma	3214	2	0.062	0.008–0.225
Endometrial cancer	1668	2	0.120	0.015–0.432
**B) FoundationCore**				
**Cancer Type**	**Total number of samples**	**Number of H773dup mutations**	**Prevalence (%)**	**Co-occurring *EFGR* amplification (%)**
Lung adenocarcinoma	33096	24	0.1	33.33
Brain glioblastoma	6162	7	0.1	42.86
Lung NSCLC (Nos)	7197	5	0.1	20.00
Bladder urothelial (transitional cell) carcinoma	3789	4	0.1	0
Kidney urothelial carcinoma	603	2	0.3	100.00
Breast phyllodes tumor	88	2	2.3	0
Lung squamous cell carcinoma	8117	2	0.02	0
Ureter urothelial carcinoma	293	1	0.3	0
Kidney renal papillary carcinoma	342	1	0.3	0
Ovary serous carcinoma	7216	1	0.01	100.00
Lung typical carcinoid	72	1	1.4	0
Brain anaplastic astrocytoma	887	1	0.1	0
Uterus endometrial adenocarcinoma endometrioid	1854	1	0.1	0

AACR, American Association for Cancer Research; EGFR, epidermal growth factor receptor; GENIE, Genomics Evidence Neoplasia Information Exchange; NSCLC, non-small cell lung cancer; Nos, not otherwise specified.

The discrepancy in the results of *EGFR* mutation testing in 2014 and 2015 may at least partly reflect the fact that Patient 1 had a multifocal adenocarcinoma, as high rates of discordance in *EGFR* mutation status and subtype have been observed between GGO lesions from the same patient (25, 26). Alternatively, this could be attributable to emergence of a secondary *EGFR* mutation after chemotherapy. The clinical implications of such spatial and temporal heterogeneity are that the use of multiple biopsies on multiple lesions and repeated biopsies upon progression, together with the use of sensitive techniques such as NGS, may be needed to fully characterize the molecular pathology of multifocal adenocarcinomas before and after treatment.

Afatinib was generally well tolerated, and diarrhea adverse events (AEs) were managed effectively with dose reductions to 20 mg/day, enabling Patient 1 to remain on afatinib treatment while still experiencing clinical benefit. This finding is consistent with previous reports that tolerability-guided dose reductions of afatinib are effective in mitigating drug-related AEs, without compromising efficacy (27).

*EGFR* exon 20 insertions are highly variable in position and size, with structural heterogeneity having implications for response to EGFR TKIs (3, 28). An *in-silico* modelling study predicted that insertions between codons 769 and 775 may be resistant to currently available EGFR TKIs, while insertions proximal to codon 769 are predicted to retain sensitivity (3). Additionally, it appears that different genomic variants confer heterogeneity in response to different EGFR-targeted therapies. Hirano et al. performed MTS assays using cells harboring four different types of *EGFR* exon 20 insertions. Afatinib potently inhibited the growth of cells harboring *EGFR* A763_Y764insFQEA (IC_50_ 3 nM vs 44 nM for osimertinib), and afatinib and osimertinib showed similar efficacy against Y764_V765insHH (134 vs 237 nM), A767_V769dupASV (158 vs 333 nM), and 770_N771insNPG (43 vs 42 nM) (16). In contrast, erlotinib and rociletinib were relatively ineffective against these mutations (16). Our own preclinical findings also demonstrated heterogeneous responses, with exon 20 mutations at different amino acid positions showing a range of sensitivities to afatinib, while being largely insensitive to erlotinib (example in **Figure 1**). A retrospective analysis of Chinese patients, in which 85 unique *EGFR* exon 20 insertion variants were identified in 547 cases (of 24,468 patients screened) indicated heterogeneous response to EGFR TKIs in the clinic. PFS differed significantly among six representative *EGFR* exon 20 insertion variants (p=0.017) with p.A763_Y764insFQEA associated with better PFS than other insertions. Afatinib and osimertinib were associated with higher disease control rate than first-generation TKIs (21).

Emerging clinical evidence further supports the hypothesis that some exon 20 insertion mutations may be sensitive to afatinib. Among patients treated with afatinib as part of the global Named Patient Use (NPU) program, 100/723 patients with any *EGFR* mutations had uncommon mutations, including 20 patients with exon 20 insertions (20). The ORR among these patients was 35% (vs 23.4% in the overall NPU population). Cases have also been reported of patients with *EGFR* exon 20 insertions responding to afatinib. In one report, two patients with *de novo EGFR* exon 20 insertions (D770_N771insSVD and Ser768_Asp770dup) showed rapid clinical improvement after afatinib treatment, but disease progression quickly ensued in one patient (29). Although the authors concluded that responses to afatinib could be short lived in some patients (29), another case study of a patient with an A767_S768insSVA tandem duplication demonstrated a durable response to afatinib, with the patient surviving for over 3 years from the start of treatment (15). In a separate report, one patient with exon 20 insertion (initially H773_V774insH, D770_N771insG, V769_D770insASV, D770_N771insSVD) was treated with osimertinib, but mutation testing following progression suggested that the mutation site had changed to A767delinsASVD only. The patient subsequently received afatinib treatment, during which the primary tumor regressed and pleural effusion was significantly reduced, with a PFS of 7.4 months (13).

An alternative strategy that has been tested against tumors with *EGFR* exon 20 insertions is to combine EGFR TKIs with anti-EGFR monoclonal antibody treatment. Preclinical evaluation of afatinib or osimertinib plus cetuximab demonstrated a mild but statistically significant additive antitumor effect of these combinations against several *EGFR* exon 20 insertion mutations *in vitro*. Afatinib plus cetuximab also significantly inhibited the growth of tumors harboring *EGFR* A767_V769dupASV and *EGFR* Y764_V765insHH, *in vivo*, while single-agent treatments did not (30). With regard to clinical data, among four patients with *EGFR* exon 20 insertions treated with afatinib plus cetuximab in the Netherlands, three patients had a partial response (PR), and the median PFS was 5.4 months (31).

Unlike *EGFR* exon 20 insertions, the spectrum of *HER2* exon 20 mutations in NSCLC is narrower, with A775_G776insYVMA accounting for most cases (18). Nevertheless, as with *EGFR* exon 20 insertions, similar heterogeneity in responses of different *HER2* exon 20 insertions to afatinib has been reported. One study investigating specific *HER2* exon 20 insertions in a Chinese cohort found that patients with tumors harboring G778_P780dup achieved numerically longer median PFS (10 vs 3.3 months, p=0.32) and overall survival (19.7 vs 7 months, p=0.16) with afatinib versus non-G778 patients, which is consistent with *in vitro* results suggesting that Glycine778 may facilitate inhibitor binding to HER2 (32). Among patients who received afatinib in a global NPU program, 12 patients with information available on the type of *HER2* mutation had an exon 20 mutation, among whom 10 patients (83%) had A775_G776insYVMA (33). Four of these patients remained on afatinib for more than 1 year, and this subgroup demonstrated a median time to treatment failure of 9.6 months, compared with just 1.9 months in the other two patients, both with M774 duplications.

Preclinical studies also suggest that irreversible EGFR TKIs such as afatinib and dacomitinib are active against *HER2* exon 20 insertions, but at ~100-fold higher concentrations than are necessary to inhibit Del19 or L858R models (34, 35). Consequently, Costa et al. tested intermittent pulsatile doses of afatinib in preclinical models of NSCLC with *HER2* exon 20 insertions, with the aim of achieving intermittent plasma concentrations that would exceed the threshold for efficacy, while improving tolerability versus daily dosing. Pulse afatinib induced anti-tumor activity in these models, and evidence of clinical activity (one PR and one stable disease) was observed among three patients with advanced *HER2* exon 20 insertion-mutated NSCLC treated with off-label pulse afatinib (36). Overall, there is accumulating evidence that afatinib may provide a viable therapeutic option for patients with at least some types of *EGFR* and *HER2* exon 20 insertion, with different approaches having been evaluated in this difficult-to-treat population.

Regarding other treatment options, a Phase 2 trial of poziotinib did not meet its primary endpoint, however the trial is ongoing in other cohorts: treatment-naïve NSCLC patients with exon 20 insertions and alternative dosing regimens to improve tolerability (9). However, despite preliminary activity, a request for breakthrough therapy designation for poziotinib for the treatment of *EGFR* exon 20 insertion mutation-positive NSCLC was rejected by the FDA. Currently, there are no treatments approved in this particular indication, although other TKIs designed to target exon 20 insertions, such as TAK-788, which has received breakthrough therapy designation from the FDA, are in early clinical development (37).

At the time that afatinib treatment was initiated in our patient, there was a lack of investigational treatments for patients with *EGFR* exon 20 insertions, and chemotherapy was and still is the standard treatment choice for these patients. In our case, the patient relapsed shortly after adjuvant chemotherapy, and afatinib treatment was chosen after detection of an exon 20 insertion mutation due to its broad inhibitory profile against uncommon *EGFR* mutations.

In conclusion, our report describes two rare cases of patients with H773dup, one of whom was treated with afatinib for 4.5 years (still on treatment at the time of this report). Our findings are in line with epidemiological evidence that this very rare (~0.1% prevalence in NSCLC), albeit widespread (across tumor types), mutation has a functional role as a driver mutation in NSCLC and can be treated with appropriate EGFR-targeted therapy. Finally, the long time on treatment and durable stable disease observed in Patient 1 is testament to afatinib’s manageable safety profile, and suggests that afatinib may be a viable therapeutic option for patients with tumors harboring this exon 20 insertion mutation, particularly those for whom chemotherapy is unsuccessful. Together with previous preclinical and clinical evidence supporting afatinib’s activity against certain *EGFR* exon 20 insertions, these findings warrant further investigation.

## Data Availability Statement

The original contributions presented in the study are included in the article/supplementary material, further inquiries can be directed to the corresponding author/s.

## Ethics Statement

Ethical review and approval was not required for the study on human participants in accordance with the local legislation and institutional requirements. The patients/participants provided their written informed consent to participate in this study. Written informed consent was obtained from both participants for the publication of this case report and any potentially identifying information/images.

## Author Contributions

SZM, BK, MB, and LM collected and assembled the data, analyzed and interpreted the data, and drafted the manuscript. HP analyzed and interpreted the data, and drafted the manuscript. AC conceived and designed the study, analyzed and interpreted the data, and drafted the manuscript. FS conceived and designed the study, collected, assembled, analyzed and interpreted the data, and drafted the manuscript. All authors provided final approval of the manuscript and agreed to be accountable for all aspects of the work, which includes ensuring that questions related to the accuracy or integrity of any part of the work are appropriately investigated and resolved. All authors contributed to the article and approved the submitted version.

## Funding

The authors would like to acknowledge the American Association for Cancer Research and its financial and material support in the development of the AACR Project GENIE registry, as well as members of the consortium for their commitment to data sharing. Interpretations are the responsibility of the study authors. Medical writing assistance, supported financially by Boehringer Ingelheim, was provided by Fiona Scott, PhD, of GeoMed, an Ashfield company, part of UDG Healthcare plc, during the preparation of this article.

## Conflict of Interest

SZM reports advisory council or committee relationship with Boehringer Ingelheim, Roche, MSD, BMS, Takeda, and AstraZeneca, honoraria from Bayer and Pfizer, and grants or funds from MSD. HP reports advisory council or committee relationship with Boehringer Ingelheim, Roche, MSD, and honoraria from Boehringer Ingelheim, Roche, MSD, BMS, and AstraZeneca. AC, FS, and MB report employment with Boehringer Ingelheim. LM reports consulting fees (advisory boards) from Boehringer Ingelheim.

The authors declare that this case report received funding for medical writing assistance from Boehringer Ingelheim. The funder was involved in the conception and design, analysis and interpretation of data, knowledge generation from molecular data, the writing and reviewing of this article, the final approval, and the decision to submit it for publication.

The remaining authors declare that the research was conducted in the absence of any commercial or financial relationships that could be construed as a potential conflict of interest.
